# *Pappa2* deletion in mice affects male but not female fertility

**DOI:** 10.1186/s12958-015-0108-y

**Published:** 2015-09-29

**Authors:** Julian K. Christians, Avery Y. King, Monika D. Rogowska, Sonia M. Hessels

**Affiliations:** Department of Biological Sciences, Simon Fraser University, 8888 University Drive, Burnaby, BC V5A 1S6 Canada

**Keywords:** Pappalysin-2, Insulin-like growth factor, Insulin-like growth factor binding protein, Ovary, Fertility

## Abstract

**Background:**

Recent studies have found associations between the gene encoding pregnancy associated plasma protein-A2 (PAPP-A2), a protease of insulin-like growth factor binding protein -5 (IGFBP-5), and measures of female reproductive performance in cattle. The purpose of the present study was to test the effects of *Pappa2* deletion on reproduction in mice.

**Findings:**

We measured the fertility and offspring growth of *Pappa2* deletion females, and also performed reciprocal matings (i.e., deletion males mated to control females) to control for the effects of offspring genotype. Ovarian and testicular IGFBP-5 levels were measured by Western blotting. As expected, deletion of *Pappa2* increased ovarian IGFBP-5 levels. However, *Pappa2* deletion in females had no effect on the interval between pairing and the birth of the first litter, the interval between the births of the first and second litters, or litter size. Offspring weight was lower in the offspring of *Pappa2* deletion females, but effects of similar magnitude were observed in the offspring of *Pappa2* deletion males, suggesting that the effects were due to heterozygosity for the deletion in the offspring. *Pappa2* deletion in males had no effect on litter size or the interval between pairing and the birth of the first litter. However, the interval between the births of the first and second litters was significantly longer in deletion males.

**Conclusions:**

*Pappa2* deletion had no effect on female reproductive performance. In contrast, *Pappa2* deletion had subtle effects on male fertility, although the underlying mechanism remains to be elucidated.

## Findings

### Background

Insulin-like growth factors (IGFs) and their binding proteins (IGFBPs) contribute to the regulation of ovarian function in mammals [1]. IGFBPs, in turn, are regulated by proteolysis [2]. Pregnancy associated plasma protein-A2 (PAPP-A2) is a protease of IGFBP-5 [3] that has been studied in the contexts of pregnancy [4–7] and postnatal growth [8–10], but not ovarian function. However, a number of recent studies have found associations between the bovine PAPP-A2 gene and rebreeding interval [11, 12], pregnancy rate [13], and age at first and second calving [14]. Furthermore, a paralog of PAPP-A2, PAPP-A, contributes to proteolytic degradation of IGFBP-2, IGFBP-4 and IGFBP-5 that is associated with follicular growth, whereas increased intrafollicular levels of IGFBPs are associated with atresia [1]. Litter size is reduced in both *Pappa* deletion mice [15] and mice overexpressing *Igfbp5* [16] suggesting that PAPP-A2 may play a role in fertility as well. In addition to its potential roles in ovarian function, PAPP-A2 has been associated with milk yield in cattle [13]. PAPP-A2 might influence lactation performance since IGFBP-5, a PAPP-A2 substrate, plays a role in mammary gland development [17], and mammary-specific expression of *Igfbp5* reduced milk production in mice [18].

To investigate the roles of PAPP-A2 in fertility and lactation, we examined the effects of *Pappa2* gene deletion in mice. *Pappa2* deletion mice are smaller than wild-type [9], and since the offspring of deletion females and control males would be heterozygous for the deletion, we expected them to be smaller than the offspring of control females and males. We therefore also examined reciprocal matings (i.e., deletion males and control females) to control for the effect of offspring genotype, independent of the maternal genotype. Since PAPP-A2 is a protease of IGFBP-5, we also tested whether *Pappa2* deletion increases the ovarian levels of IGFBP-5, as it does the circulating levels of IGFBP-5 [19].

### Methods

#### Pappa2 deletion mice

All work was carried out in accordance with the guidelines of the Canadian Council on Animal Care and approved by the SFU University Animal Care Committee (protocol 1035B-11). *Pappa2* deletion mice with a C57BL/6 background were generated as previously described [9, 19] The intact conditional allele (*Pappa2*^*fl*^) was used as the control for the deletion allele (*Pappa2*^*KO*^); there is no difference in postnatal weight gain between mice homozygous for the *Pappa2*^*fl*^ allele and littermates homozygous for the wild-type allele [19]. Mice were ear-clipped at weaning and PCR genotyping was performed as previously described [19].

#### Matings

Females or males homozygous for the deletion allele (*Pappa2*^*KO/KO*^) or the intact conditional allele (*Pappa2*^*fl/fl*^) were paired with mice with two intact alleles. Following pairing, cages were checked every morning, and newborn pups were counted. Litters with evidence of cannibalization were included in analyses of time to birth, but not in analyses of litter size. At three weeks pups were sexed and weighed.

#### Western blotting

Virgin mice were culled at approximately 19 weeks of age (i.e., the age at which the mice in the breeding experiments were paired) to collect ovaries and testes. Protein extraction and Western blotting were performed as described previously [9] using a 12.5 % polyacrylamide gel for separation and a primary antibody solution containing 1:1000 monoclonal mouse anti-actin (CLT9001; Cedarlane, Burlington ON) and 1:500 polyclonal goat-anti mouse IGFBP-5 (AF578; R&D Systems). Membranes were visualized using the Odyssey infrared imaging system (Li-Cor Biosciences, Lincoln, NE) which allowed simultaneous quantification of IGFBP-5 and actin.

### Results and discussion

#### Female fertility

**Fig. 1:**
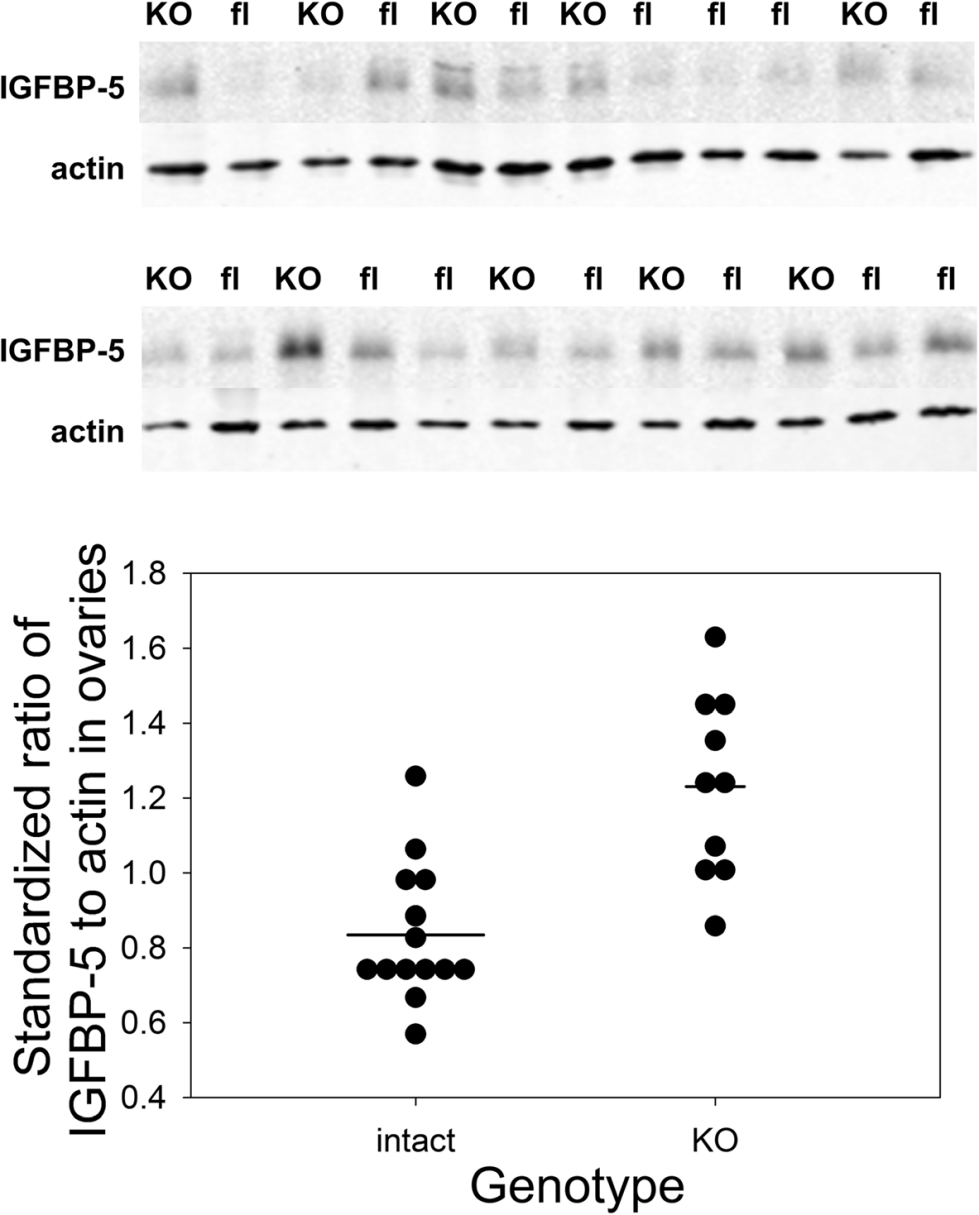
Ovarian IGFBP-5 levels, measured by Western blotting. Upper: Western blotting of IGFBP-5 and actin in the ovaries of females homozygous for the deletion allele (*Pappa2* ^*KO/KO*^; KO) or the intact conditional allele (*Pappa2* ^*fl/fl*^; fl). Lower: Ratio of the intensity of the IGFBP-5 band to that of the actin band, standardized by dividing an individual sample’s ratio by the mean ratio of all samples on the same blot, such that the average value is 1. Horizontal lines represent the means for each group

As expected, deletion of *Pappa2* increased ovarian levels of IGFBP-5 (F_1,22_ = 20.89, P < 0.0001; Fig. 1). However, all pairs gave birth to at least one litter, and there was no effect of *Pappa2* deletion in females on the interval between pairing and the birth of the first litter, the interval between the births of the first and second litters, or litter size (Table 1). The lack of an effect of *Pappa2* deletion is in contrast to the reduced litter size observed in *Pappa* deletion mice [15], suggesting different roles for IGFBP-4 (a substrate of PAPP-A, but not PAPP-A2) and IGFBP-5 (a substrate of both proteases) [3, 15] in ovarian function. Indeed, in mice it is thought that IGFBP-4 may induce atresia of follicles whereas IGFBP-5 expression decreases with follicular atresia [20]. While *Pappa2* deletion was sufficient to elevate IGFBP-5 protein levels in whole ovaries, it was not sufficient to mimic the effects of transgenic *Igfbp5* overexpression on fertility [16].

**Table 1:** Reproductive traits of mice homozygous for *Pappa2* disruption (*Pappa2* ^*KO/KO*^) or homozygous for the intact allele (*Pappa2* ^*fl/fl*^)

	Females			Males		
	*Pappa2* ^*fl/fl*^	*Pappa2* ^*KO/KO*^	P-value^a^	*Pappa2* ^*fl/fl*^	*Pappa2* ^*KO/KO*^	*P*-value^a^
N	15	17		6	10	
Mass at pairing (g)	23.5 ± 0.5	20.7 ± 0.4	0.0001	30.5 ± 0.7	24.6 ± 0.5	0.0001
Age at pairing (weeks)	19.5 ± 0.4	19.4 ± 0.4	0.80	19.5 ± 0.8	19.4 ± 0.6	0.97
Days between pairing and birth of first litter	27.0 ± 2.0	24.0 ± 1.9	0.28	25.3 ± 2.2	22.3 ± 1.7	0.28
Days between births of first and second litters	30.6 ± 1.1	26.7 ± 1.1	0.11	23.1 ± 1.1	38.8 ± 1.1	0.0001
Proportion of matings that produced second litter within 35 days of birth of first litter^b^	8/14^c^ = 57.14 %	15/17 = 88.24 %	0.10	6/6 = 100 %	2/10 = 20 %	0.0070
Size of first litter at birth	8.1 ± 0.6	7.8 ± 0.6	0.75	8.8 ± 0.7	8.4 ± 0.6	0.65
Size of first litter at wean	7.5 ± 0.6	7.3 ± 0.6	0.85	8.6 ± 0.8	8.2 ± 0.6	0.69
Size of second litter at birth	7.6 ± 0.6	8.1 ± 0.5	0.50	6.3 ± 0.9	8.2 ± 1.0	0.20
Size of second litter at wean	6.9 ± 0.6	7.4 ± 0.5	0.54	5.8 ± 0.8	6.0 ± 0.9	0.90

^a^For most traits, values are least squares means ± standard error from a general linear model including the effect of genotype (GLM procedure; SAS, ver 9.3). The number of days between the births of the first and second litters was analysed using failure time analysis with a log-logistic distribution, with females that did not produce a second litter included as right-censored observations (LIFEREG procedure), and the proportion of matings that produced a second litter was analysed using Fisher’s Exact Test (FREQ procedure, chisq option). Analyses with nonparametric tests (Wilcoxon, Kruskal-Wallis; NPAR1WAY procedure) yielded qualitatively similar results

^b^In the first set of pairs, males were removed from females two weeks after the first litter was born, and therefore the females could only have given birth to a second litter within 35 days of the birth of the first litter. In contrast, pairs in the second set of matings were left together until the birth of the second litter

^c^One female died while delivering first litter and so was not included in this analysis

#### Offspring size

**Fig. 2:**
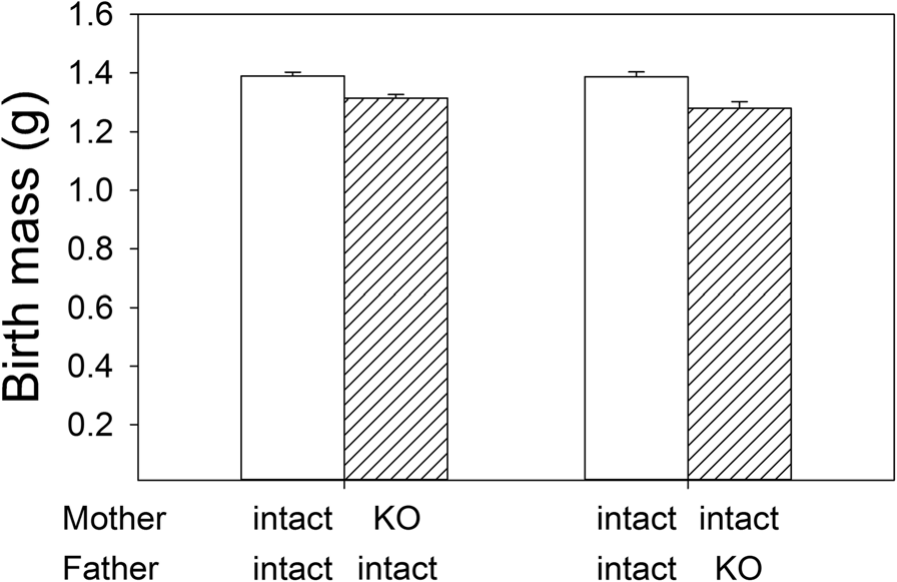
Effects of *Pappa2* deletion on birth weight of offspring. Values are least squares means ± standard error from a repeated measures analysis (MIXED procedure; SAS, ver 9.3) including pair as the subject and the effects of genotype and parity (first or second litter). Repeated measures analysis was used since there were multiple pups for each female. Birth weights were only measured in some pairs, so the total sample size was 17 pairs (187 pups) for the analysis of female genotype, and 9 pairs (96 pups) for male genotype

Birth weight and the weight of offspring at three weeks of age were analysed using repeated measures analyses since there were multiple pups for each female. Deletion of *Pappa2* in the female reduced offspring weight at birth (F_1,15_ = 17.92, P = 0.0007; Fig. 2), but there were no effects of parity (i.e., first vs. second litter; F_1,8_ = 4.40, P = 0.07). However, the effect of genotype was also significant when *Pappa2* was deleted in males (F_1,7_ = 16.42, P = 0.005; Fig. 2; effect of parity: F_1,3_ = 1.88, P = 0.26), suggesting that the effect was due to offspring and not parental genotype. Offspring of deletion parents were heterozygous for the deletion, whereas offspring of controls were homozygous for an intact allele. Although the effects of *Pappa2* deletion are partially recessive, mice heterozygous for the *Pappa2* deletion are slightly lighter than wild-type homozygotes [19].

**Fig. 3:**
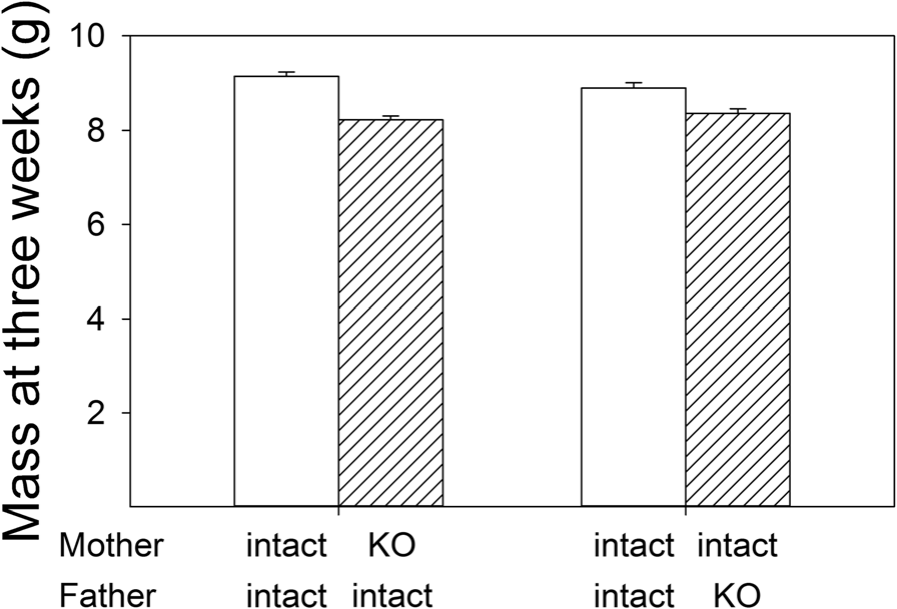
Effects of *Pappa2* deletion on the weight of offspring at three weeks of age. Values are least squares means ± standard error from a repeated measures analysis (MIXED procedure; SAS, ver 9.3) including pair as the subject and the effects of genotype, parity (first or second litter) and offspring sex. The total sample size was 29 pairs (357 pups) for the analysis female genotype, and 16 pairs (190 pups) for male genotype

As with birth weight, offspring weight at three weeks of age was reduced by *Pappa2* deletion in the female (F_1,27_ = 56.58, P < 0.0001; Fig. 3) or in the male (F_1,14_ = 12.71, P = 0.0031; Fig. 3), again suggesting that the effect was due to the genotype of the offspring. These analyses also included effects of parity and offspring sex, which were significant for analyses of *Pappa2* deletion in females (parity: F_1,19_ = 8.48, P = 0.0089; sex: F_1,27_ = 18.52, P = 0.0002) and males (parity: F_1,9_ = 11.32, P = 0.0083; sex: F_1,14_ = 20.59, P = 0.0005), with male offspring being heavier than female offspring. These results suggest that the deletion of *Pappa2* did not increase IGFBP-5 levels sufficiently to achieve the reduced milk production observed in transgenic mice overexpressing *Igfbp5* in the mammary gland [18].

#### Male fertility

Because we examined reciprocal matings to control for the effect of offspring genotype, we were also able to examine the effects of *Pappa2* deletion on male fertility. While there was no effect on litter size or the interval between pairing and the birth of the first litter, *Pappa2* deletion in males significantly increased the interval between the births of the first and second litters (Table 1). This result is from a failure-time analysis that included females that did not produce a second litter as right-censored observations. Therefore, this result could reflect females mated to *Pappa2* deletion males (a) taking longer to become pregnant, (b) being less likely to become pregnant a second time, or (c) having a higher probability of fetal resorption or spontaneous abortion. Females mated to *Pappa2* deletion males were less likely to become pregnant within two weeks of the birth of the first litter (and so give birth to a second litter within 35 days of the birth of the first litter; Table 1). However, even among females who did give birth to a second litter within 35 days of the birth of the first litter, there was a trend whereby females mated to *Pappa2* deletion males tended to have a longer interval between first and second litters (F_1,6_ = 4.29, P = 0.08). It is not clear why *Pappa2* deletion in males affected the interval between first and second litters but not the interval between pairing and the birth of the first litter, although males may have become more susceptible to the effects of *Pappa2* deletion as they aged.

**Fig. 4:**
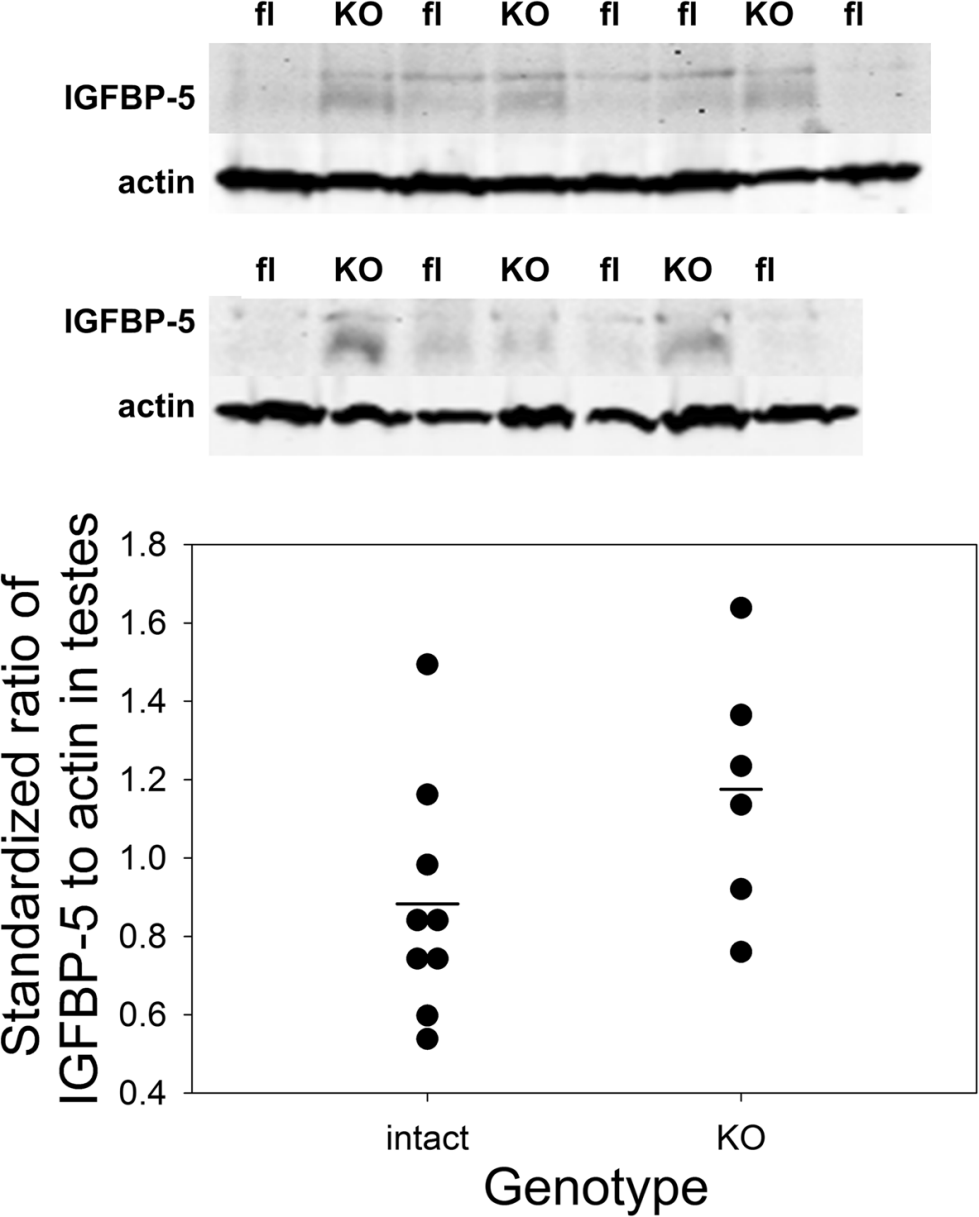
Testicular IGFBP-5 levels, measured by Western blotting. Upper: Western blotting of IGFBP-5 and actin in the testes of males homozygous for the deletion allele (*Pappa2* ^*KO/KO*^; KO) or the intact conditional allele (*Pappa2* ^*fl/fl*^; fl). Lower: Ratio of the intensity of the IGFBP-5 band to that of the actin band, standardized by dividing an individual sample’s ratio by the mean ratio of all samples on the same blot. Horizontal lines represent the means for each group

The effect of *Pappa2* deletion in males, i.e., increased time between litters without an effect on litter size, is similar to that of prostate removal [21]. Since the PAPP-A2 substrate, IGFBP-5, is associated with decreased proliferation and/or increased apoptosis in the prostate [22–24], *Pappa2* deletion would be expected to increase local IGFBP-5 levels and so decrease proliferation/increase apoptosis in the prostate, leading to subfertility. IGFBP-5 in the prostate was not detectable by Western blotting (data not shown), but was detectable in the testes, and tended to be increased by *Pappa2* deletion, but the difference was not significant (F_1,13_ = 3.36, P = 0.09; Fig. 4). While the influence of PAPP-A2 on male fertility may involve IGFBP-5 and the regulation of IGF availability, it has recently been suggested that PAPP-A2 may also act through IGFBP-5 independent pathways [25].

### Conclusions

*Pappa2* deletion had no effect on female fertility, in contrast to the effects of *Pappa* deletion or *Igfbp5* overexpression. Deletion of *Pappa2* had subtle effects on male fertility, increasing the interval between first and second litters but not the interval between pairing and the birth of the first litter. The mechanism underlying this effect on male fertility remains to be elucidated.

## Abbreviations

IGF: Insulin-like growth factor
IGFBP: Insulin-like growth factor binding protein
PAPP-A: Pregnancy-associated plasma protein–A
SNP: Single nucleotide polymorphism
